# State of the art and perspectives of gene therapy in heart failure. A scientific statement of the Heart Failure Association of the ESC, the ESC Council on Cardiovascular Genomics and the ESC Working Group on Myocardial & Pericardial Diseases

**DOI:** 10.1002/ejhf.3516

**Published:** 2024-11-22

**Authors:** Sophie Van Linthout, Konstantinos Stellos, Mauro Giacca, Edoardo Bertero, Antonio Cannata, Lucie Carrier, Pablo Garcia‐Pavia, Alessandra Ghigo, Arantxa González, Kristina H. Haugaa, Massimo Imazio, Luis R. Lopes, Patrick Most, Piero Pollesello, Heribert Schunkert, Katrin Streckfuss‐Bömeke, Thomas Thum, Carlo Gabriele Tocchetti, Carsten Tschöpe, Peter van der Meer, Eva van Rooij, Marco Metra, Giuseppe M.C. Rosano, Stephane Heymans

**Affiliations:** ^1^ Berlin Institute of Health (BIH) at Charité – Universitätmedizin Berlin BIH Center for Regenerative Therapies (BCRT) Berlin Germany; ^2^ German Center for Cardiovascular Research (DZHK) partner site Berlin Berlin Germany; ^3^ Department of Cardiovascular Research, Medical Faculty Mannheim Heidelberg University Mannheim Germany; ^4^ Department of Cardiology, Angiology, Haemostaseology and Medical Intensive Care University Medical Centre Mannheim, Heidelberg University Mannheim Germany; ^5^ German Centre for Cardiovascular Research (DZHK) partner site Heidelberg/Mannheim Mannheim Germany; ^6^ Helmholtz Institute for Translational AngioCardioScience (HI‐TAC) Mannheim Germany; ^7^ Biosciences Institute, Vascular Biology and Medicine Theme, Faculty of Medical Sciences Newcastle University Newcastle UK; ^8^ School of Cardiovascular and Metabolic Medicine & Sciences and British Heart Foundation Centre of Research Excellence, King's College London, London, UK; Department of Medical Sciences University of Trieste Trieste Italy; ^9^ Cardiovascular Unit, Department of Internal Medicine University of Genova Genova Italy; ^10^ School of Cardiovascular and Metabolic Medicine & Sciences and British Heart Foundation Centre of Research Excellence King's College London London UK; ^11^ Department of Experimental Pharmacology and Toxicology University Medical Center Hamburg‐Eppendorf Hamburg Germany; ^12^ German Centre for Cardiovascular Research (DZHK) partner site Hamburg/Kiel/Lübeck Hamburg Germany; ^13^ Hospital Universitario Puerta de Hierro Majadahonda, IDIPHISA, CIBERCV Madrid Spain; ^14^ Centro Nacional de Investigaciones Cardiovasculares (CNIC) Madrid Spain; ^15^ Universidad Francisco de Vitoria (UFV) Madrid Spain; ^16^ Department of Molecular Biotechnology and Health Sciences Molecular Biotechnology Center "Guido Tarone," University of Torino Torino Italy; ^17^ Program of Cardiovascular Diseases, CIMA and Department of Pathology, Anatomy and Physiology Universidad de Navarra Pamplona Spain; ^18^ IdiSNA Navarra Institute for Health Research Pamplona Spain; ^19^ CIBERCV (Network for Biomedical Research in Cardiovascular Disease) Instituto de Salud Carlos II Madrid Spain; ^20^ ProCardio Center for Innovation, Department of Cardiology Oslo University Hospital, Rikshospitalet Oslo Norway; ^21^ Faculty of Medicine, Institute of Clinical Medicine University of Oslo Oslo Norway; ^22^ Department of Medicine (DMED), University of Udine, and Cardiothoracic Department ASUFC University Hospital Santa Maria della Misericordia Udine Italy; ^23^ Institute of Cardiovascular Science University College London London UK; ^24^ Barts Heart Centre, St Bartholomew's Hospital London UK; ^25^ Department of Cardiology, Angiology, Pulmonology University Hospital Heidelberg Heidelberg Germany; ^26^ Content and Communication, Branded Products Espoo Finland; ^27^ Department of Cardiology, Deutsches Herzzentrum München Technische Universität München Munich Germany; ^28^ German Center for Cardiovascular Research (DZHK) Partner Site Munich Heart Alliance Munich Germany; ^29^ Clinic for Cardiology and Pneumology University Medical Center Göttingen Germany; ^30^ German Center for Cardiovascular Research (DZHK), Partner site Göttingen Göttingen Germany; ^31^ Institute of Pharmacology and Toxicology University of Würzburg Würzburg Germany; ^32^ Department of Translational Research, Comprehensive Heart Failure Center (CHFC) University Clinic Würzburg Würzburg Germany; ^33^ Institute of Molecular and Translational Therapeutic Strategies (IMTTS) Hannover Medical School Hannover Germany; ^34^ Department of Translational Medical Sciences; Center for Basic and Clinical Immunology Research (CISI); Interdepartmental Center for Clinical and Translational Research (CIRCET); Interdepartmental Hypertension Research Center (CIRIAPA) Federico II University Naples Italy; ^35^ Deutsches Herzzentrum der Charité (DHZC), Department of Cardiology, Angiology and Intensive Medicine Campus Virchow Klinikum Berlin Germany; ^36^ Department of Cardiology University Medical Center Groningen, University of Groningen Groningen The Netherlands; ^37^ Hubrecht Institute Royal Netherlands Academy of Arts and Sciences (KNAW) and University Medical Center Utrecht Utrecht The Netherlands; ^38^ Department of Cardiology University Medical Center Utrecht Utrecht The Netherlands; ^39^ Cardiology, ASST Spedali Civili di Brescia, Department of Medical and Surgical Specialties, Radiological Sciences, and Public Health University of Brescia Brescia Italy; ^40^ Cardiovascular Clinical Academic Group, St. George's University Hospitals, NHS Trust University of London London UK; ^41^ Cardiology, San Raffaele Cassino Hospital Cassino Italy; ^42^ Department of Human Sciences and Promotion of Quality of Life San Raffaele University of Rome Rome Italy; ^43^ Centre for Molecular and Vascular Biology KU Leuven Leuven Belgium; ^44^ Department of Cardiology Maastricht University, CARIM School for Cardiovascular Diseases Maastricht The Netherlands; ^45^ European Reference Network for Rare Low Prevalence and Complex Diseases of the Heart (ERN GUARD‐Heart) Amsterdam The Netherlands

**Keywords:** Adeno‐associated viral vector, Gene editing, Gene replacement, Gene silencing, Gene therapy, Heart failure

## Abstract

Gene therapy has recently become a reality in the treatment of cardiovascular diseases. Strategies to modulate gene expression using antisense oligonucleotides or small interfering RNA are proving to be safe and effective in the clinic. Adeno‐associated viral vector‐based gene delivery and CRISPR‐Cas9‐based genome editing have emerged as efficient strategies for gene delivery and repair in humans. Overall, gene therapy holds the promise not only of expanding current treatment options, but also of intervening in previously untackled causal disease mechanisms with little side effects. This scientific statement provides a comprehensive overview of the various modalities of gene therapy used to treat heart failure and some of its risk factors, and their application in the clinical setting. It discusses specifically the possibilities of gene therapy for hereditary heart diseases and (non)‐genetic heart failure. Furthermore, it addresses safety and clinical trial design issues and challenges for future regulatory strategies.

## Introduction

**Figure 1:**
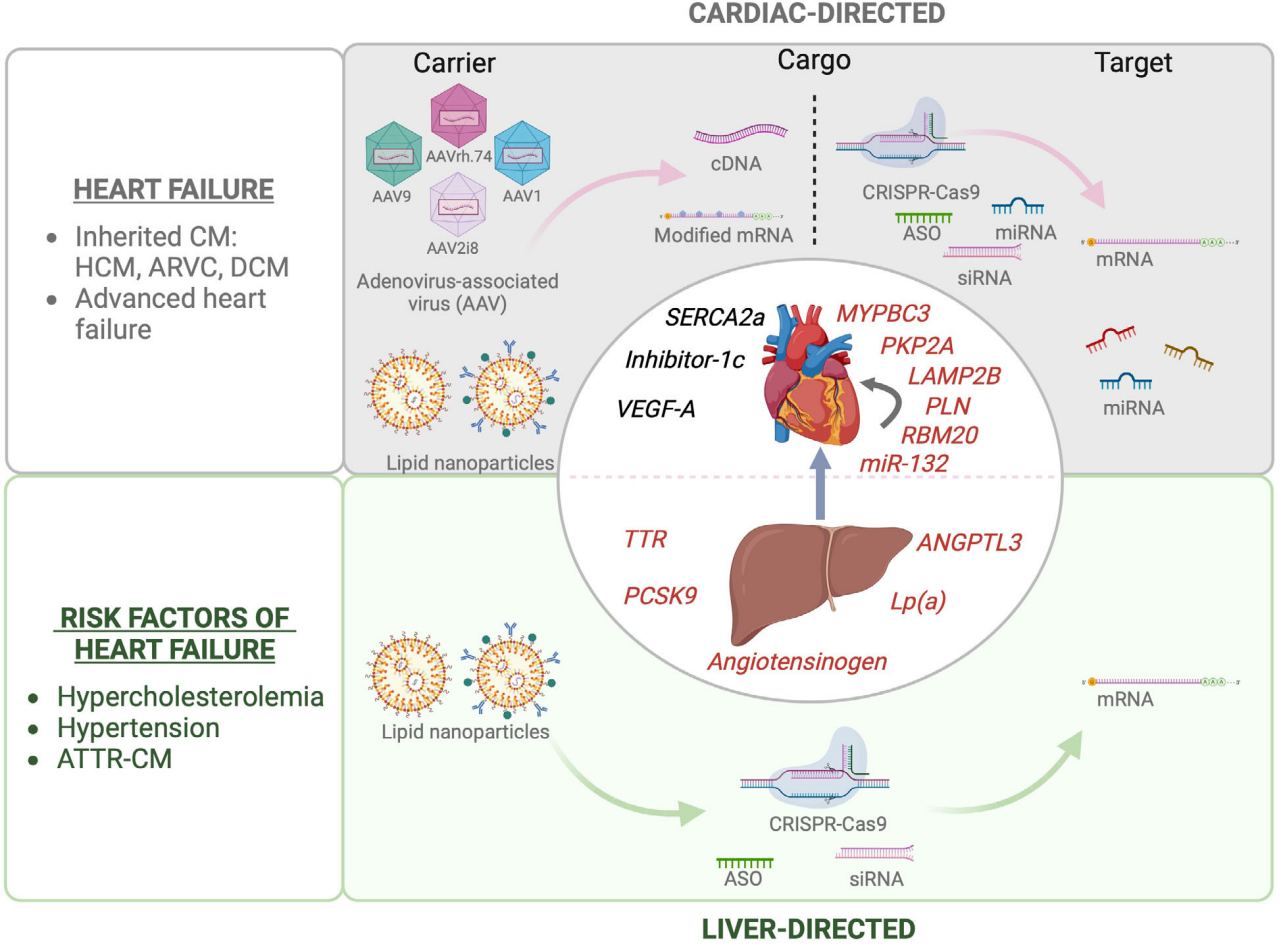
Cardiac‐ and liver‐directed gene therapy for heart failure and risk factors underlying heart failure. Main delivery methods, cargo, and targets currently used in gene therapy for heart failure and risk factors underlying heart failure distinguishing cardiac‐ and liver‐directed gene therapy with the latter mainly focused on gene silencing/gene editing strategies (ASO, siRNA, CRISPR‐Cas9) delivered via lipid nanoparticles. In contrast, cardiac gene therapy comprises adeno‐associated viral‐ and lipid nanoparticle‐mediated delivery strategies which may carry cDNA or modified mRNA, respectively, or modalities for gene silencing/editing/replacement (ASO, siRNA, miRNA, CRISPR‐Cas9). Genes/mRNA for overexpression are outlined in black, silencing/editing/replacement targets are marked in red. ANGPTL3, angiopoietin‐like 3; ARVC, arrhythmogenic right ventricular cardiomyopathy; ASO, antisense oligonucleotide; ATTR, transthyretin amyloidosis; CM, cardiomyopathy; DCM, dilated cardiomyopathy; HCM, hypertrophic cardiomyopathy; LAMP2B, lysosome‐associated membrane protein 2b; Lp(a), lipoprotein(a); MYBPC3, cardiac myosin‐binding protein C; PCSK9, proprotein convertase subtilisin/kexin type 9; PKP2A, plakophilin 2a; PLN, phospholamban; RBM20, RNA binding motif protein 20; SERCA2a, sarcoplasmic/endoplasmic reticulum Ca^2+^ ATPase 2a; siRNA; small interfering RNA; TTR, transthyretin; VEGF‐A, vascular endothelial growth factor‐A. Created in BioRender. Van Linthout S. (2024) BioRender.com/n39e845

The concept of gene therapy, a strategy to treat diseases using viral or non‐viral vectors that introduce therapeutic exogenous genes into target cells and tissue to supplement or correct defective genes, was first formally proposed by Friedmann and Roblin in the 1970s.^1^ Fifty years later, gene therapy has become a reality in treatment of cardiovascular diseases (CVD). In addition to adding genes using protein‐coding cDNAs, strategies that enable precise gene editing using clustered regularly interspaced short palindromic repeat (CRISPR)‐associated protein 9 (CRISPR‐Cas9),^2^ modulation of gene expression using RNA therapeutics (antisense oligonucleotides [ASOs], small interfering RNAs [siRNAs] and microRNAs [miRNAs])^3^, ^4^, ^5^ and of protein expression using modified mRNA (modRNA) have entered the gene therapy field. Adeno‐associated viral (AAV) vectors have become the gold standard for *in vivo* viral gene therapy,^6^ while formulations based on polymers, lipids, peptides or other nano‐ or micro‐sized particles have been developed as non‐viral carriers (*Figure* *1*).^7^ Several gene therapies for cardiac diseases have been investigated in clinical trials, but success in clinical translation has been highly dependent on delivery technologies. Clinical success has been reached primarily with liver‐directed therapies that target genes of risk factors underlying heart failure^8^, ^9^, ^10^, ^11^ or diseases in which proteins produced by the liver affect the heart and its vasculature.^12^ However, gene therapies targeting the cardiac muscle have been less successful so far.^13^, ^14^, ^15^ This scientific document provides an overview of existing gene therapy modalities and their clinical application, and discusses the opportunities and challenges of gene therapy for non‐genetic heart failure and inherited heart diseases.

## Updates on different modalities for gene therapy

### Antisense oligonucleotides and small non‐coding RNAs (siRNAs and miRNAs)

Single‐stranded ASOs and small non‐coding RNAs (siRNAs and miRNAs) share a fundamental principle: an oligonucleotide binds a target RNA by Watson–Crick base pairing. An ASO must survive and function as a single strand. In contrast, an siRNA or miRNA, delivered as a small RNA duplex, is loaded within the RNA‐induced silencing complex (RISC), whereupon one strand (the passenger strand) is discarded, and the remaining strand (the guide strand) cooperates with RISC to bind complementary RNA. This distinction between ASOs and siRNAs/miRNAs results in different strengths and weaknesses that affect drug development.^16^ Unmodified single‐stranded ASOs are too unstable to be used in cells. Their stability and pharmacological properties can be increased by incorporating chemical modifications.^3^, ^17^, ^18^

One category of ASOs targets endogenously expressed miRNAs, which have emerged as pivotal regulators in the pathogenesis of CVD, including atherosclerosis, arrhythmias, and heart failure. miRNAs modulate gene expression at the post‐transcriptional level and play a critical role in regulating vascular function, cardiomyocyte hypertrophy, inflammation, and fibrosis, all of which are central to CVD development and progression.^19^ Anti‐miRNA ASOs have already progressed to clinical trials for heart failure and other CVD.^20^

While ASOs continue to be used for gene silencing, the robust nature of siRNAs and the relative ease of identifying active siRNAs through systematic screenings have made these molecules a preferred silencing tool.

One area where miRNA therapy appears particularly exciting is cardiac regeneration, as this process requires the reprogramming of cardiomyocytes to a proliferative state, which can be achieved by miRNAs due to their ability to simultaneously target multiple cellular mRNA targets.^4^ In mice, single intramyocardial injection of the pro‐regenerative miR‐199a‐3p or miR‐590‐3p,^21^ miR‐19a/19b,^22^ miR302b/c,^23^ or miR‐708^24^ mimics, has been shown to promote cardiomyocyte proliferation, cardiomyocyte stress resistance and cardiac regeneration in response to myocardial ischaemia. Therapy with some of these miRNAs is now being investigated for efficacy and safety in large animal studies and will eventually be tested in clinical trials.

### Modified mRNAs

Modificationof structural elements of mRNA – notably the 5′ cap, 5′‐ and 3′‐UTRs, the coding region, and the poly(A) tail – has systematically improved its intracellular stability, reduced its immunogenicity (recognition via toll‐like receptors) and increased its translational efficiency.^25^, ^26^, ^27^, ^28^ These improvements ultimately have led to the production of significant levels of the encoded protein over a longer timeframe. Consequently, the use of modRNAs has emerged as an appealing therapeutic tool for protein overexpression in heart disease.^29^, ^30^, ^31^ The challenge still remains to improve modRNA delivery to achieve non‐invasive, cell‐specific delivery targeting the heart, the same challenge as with ASO, siRNA and miRNA. Moreover, protein expression is transient and repeated administration would be needed in chronic conditions.

Most research in this area has focused on ischaemic heart disease and myocardial infarction. A reduction in infarct size and improvement in cardiac function has been shown following application of modRNA for mutated human follistatin‐like 1,^32^ pyruvate kinase muscle isoform 2,^33^ yes‐associated protein,^34^ acid ceramidase,^35^ insulin growth like factor‐1^36^ and vascular endothelial growth factor‐A^37^, ^38^ in mice or pigs. The success of this strategy has prompted its translation to human clinical trials (see below).

Interestingly, modRNAs have also been used to generate transient antifibrotic chimeric antigen receptor (CAR) T cells *in vivo* targeting activated fibroblasts.^39^ Treatment with modRNA encoding a CAR against fibroblast activation protein encapsulated in CD5‐targeted lipid nanoparticles reduced fibrosis and restored cardiac function in an experimental murine heart failure model, illustrating the potential of *in vivo* generation of CAR T cells as a therapeutic platform to treat various CVD.

### Non‐viral carriers

Nucleic acids are hydrophilic, positively charged and susceptible to nuclease degradation, all of which are barriers to their delivery through cell membranes. Single‐stranded ASOs are endocytosed and can pass freely through cellular membranes when administered as naked molecules.^40^ Delivery of naked siRNA is more challenging since the RNA duplex is highly hydrophilic, making cellular permeability relatively modest. Targeting of both ASOs and siRNAs to hepatocytes is facilitated by their conjugation with N‐acetylgalactosamine, which binds the asialoglycoprotein receptor, which is highly expressed on hepatocytes.^41^ Conjugation with N‐acetylgalactosamine is the most effective delivery method today, which allows specific delivery to the liver.^41^ It is hoped that the discovery of new conjugations will allow more specific delivery to the heart and other organs or cells such as the kidney or inflammatory cells.

ModRNAs can be injected into the myocardium as naked molecules in sucrose citrate buffer,^31^, ^33^ but this requires relatively high doses. In most applications, RNA is therefore delivered using formulations based on polymers, lipids, peptides or other particles at the nano‐ or microscale.^5^ Currently, the most successful formulations are lipid nanoparticles obtained by the stable nucleic acid lipid particle technology,^7^ which were originally developed in the late 1990s, gradually improved^42^, ^43^ and popularized by the mRNA‐based COVID‐19 vaccines. This technology is further instrumental for the development of *in vivo* CRISPR/Cas9 gene editing strategies directed to the liver.^12^

### Adeno‐associated viral vectors

A family of vectors that is currently considered the gold standard for gene therapy and gene editing of the heart is based on cardiotropic serotypes of the small parvovirus AAV. AAV transduce postmitotic cells, especially cardiomyocytes, and can effectively, persistently and safely express foreign genes in host cells with low cytotoxicity and immunogenicity. Despite these unique characteristics, the ability to deliver the genetic payload (physical transduction) and achieve high transgene expression (functional transduction) remains the most important feature for the selection and improvement of AAV variants for clinical applications.^6^

As AAV capsid proteins play an essential role in delivery/tropism, AAV capsid variants have been generated for improved cardiac selectivity and liver de‐targeting. Besides variants targeting cardiomyocytes,^44^ variants based on AAV2^45^ or AAV9^46^ serotypes have also been generated to target endothelial cells. Moreover, AAV targeting cardiac fibroblasts^47^ have recently been developed for therapies to counteract fibrosis and the fibro‐inflammatory response therapeutically.^48^, ^49^ In addition to AAV‐specific targeting of cardiac cells, selective promoters and enhancers for cardiac cell types (cardiomyocyte,^50^ endothelial cell,^51^ cardiac fibroblast) are used to avoid ectopic gene expression.

### Gene editing

CRISPR/Cas9 edits genes by precisely introducing double‐stranded breaks into the DNA and then allowing natural DNA repair processes to take over. The system consists of two components: the Cas9 enzyme, which generates site‐specific double‐stranded breaks and a guide RNA, which directs the endonuclease to the site to be edited. Base editing allows the precise modification of a single base in the target site without causing double‐stranded breaks,^52^ with cytidine base editors (which convert a C‐G base pair to T‐A) and adenine base editors (which convert a A‐T to G‐C) being the two most commonly used editors so far.^53^ Finally, prime editing enables precise correction of relatively long DNA segments, using a modified guide RNA carrying the desired sequence as a template.^2^

CRISPR‐Cas9 gene editing technology is commonly used to correct specific genetic variants prior to disease onset,^2^ as shown for the treatment of inherited cardiomyopathies in experimental models, as well as in hereditary transthyretin (TTR) amyloidosis (ATTR) (ATTRv) and familial hypercholesterolaemia. Recently, CRISPR‐Cas9 adenine base editors have also been used in a murine ischaemia/reperfusion model to disrupt the pathological overactivation of calcium (Ca^2+^)/calmodulin‐dependent protein kinase IIδ, a primary driver of cardiac disease.^54^ This cardioprotective strategy is potentially applicable to a broad range of patients with already‐established heart disease. The concept of using CRISPR‐Cas9 to block the activation of harmful signalling pathways can also be translated to harmful signalling cascades in other human diseases.

## What has already reached the clinic?

Clinical trials evaluating gene therapy strategies for heart failure and risk factors underlying heart failure are outlined in *Table* *1*, which gives an overview of their clinical endpoint, safety measures and clinical side effects.

**Table 1:** Clinical trials evaluating gene therapy strategies for heart failure and for risk factors of heart failure.

Modality/target organ									
Drug/GTMP	Carrier/ target	Cardiac disorder/ risk factor	Admin. route	Duration	Clinical endpoint	Safety measures	Clinical side effect	Clinical trials	
								Phase	Name/NCT
**siRNA/liver**									
Patisiran	ATTR	ATTRv with polyneuropathy	s.c.	18 months	Improvement in neuropathy and polyneuropathy.	Clinical laboratory tests, thyroid function, anti‐drug antibodies, ECG, electroretinogram, vital signs. Premedications to reduce the likelihood of infusion‐related reactions.	Mild or moderate infusion‐related reactions. Overall incidence and types of SAE were similar in placebo and patisiran‐treated group.	3	APOLLO^55^
		ATTRv with polyneuropathy and evidence of cardiac amyloid involvement			Reduction in LV wall thickness and NT‐proBNP compared with both baseline and placebo.	Monitoring of cardiac AEs and cardiac arrhythmia AEs. Vitamin A supplementation	Cardiac failure AEs were similar in thepatisiran and placebo groups.	3	Prespecified subpopulation of APOLLO^59^
		ATTR‐CM		12 months	Preservation of functional capacity, health status and QOL.	AEs, clinical laboratory variables, vital signs	Infusion‐related reactions, arthralgia, and muscle spasms	3	APOLLO‐B,^60^ NCT03997383
Vutrisiran	ATTR	ATTRv with polyneuropathy	s.c.	18 months	Significant clinical benefits in multiple measures of QOL and physical function.	Treatment‐emergent AEs, vitamin A supplementation Patients randomized to patisiran received premedication to minimize risk of infusion‐relatedreactions	Mild or moderate AEs, no drug‐relateddiscontinuations or deaths	3	HELIOS‐A^61^
		ATTR‐CM		36 months	Lower risk of death from any cause and CV events than placebo and preservedfunctional capacity and QOL.	AEs, clinical laboratory measure, vital signs. Vitamin A supplementation.	Incidence of AEs was similar in the two groups	3	HELIOS‐B,^63^ NCT04153149
Inclisiran	PCSK9	Familial hypercholesterolaemia	s.c.	540 days	Reductions in LDL‐cholesterol levels of approximately 50% were obtained.	AEs, laboratory values, vital signs, ECG, injection‐site reactions, antidrug antibodies.	AEs and SAEs were similar in the two groups.	3	ORION‐9^8^
		Patients with elevated LDL‐cholesterol				AEs, clinical laboratory values, antidrug antibodies.	AEs were generally similar in the inclisiran and placebo groups in each trial, although injection‐site AEs were more frequent with inclisiran than with placebo. Such reactions were generally mild, and none were severe or persistent.		ORION‐10^64^; ORION‐11^64^
ARO‐ANG3	ANGPTL3	Familial hypercholesterolaemia	s.c.	16 weeks	Reduction in triglyceride and non‐HDL‐cholesterol.	AEs or SAEs, physical examinations, ECG, injection site reactions, clinicallaboratory tests levels, concomitant medications/therapy; and reasons for treatment discontinuation due to toxicity.	Well tolerated, no deaths, no life‐threateningTEAEs or TEAEs leading to drug discontinuation or premature withdrawal of any participant from the study. No treatment‐related SAEs. No adverse changes in laboratory parameters. No clinically meaningful declines in platelet count.	1	AROANG1001^178^
Olpasiran	Lp(a)	Increased Lp(a) in patients with established atherosclerotic cardiovascular disease.	s.c.	48 weeks	Significant reduction in Lp(a) concentrations.	Monitoring of AEs and safety laboratory values.	Overall incidence of AEs was similar across the trial groups. Injection‐site reactions, primarily pain	1	OCEAN‐DOSE^11^
Zilebesiran	Angioten‐sinogen	Hypertension	s.c.	24 months	Dose‐dependent decreases in serum angiotensinogen levels and 24‐hour ambulatory blood pressure.	Monitoring of AEs, laboratory assessments, and vital signs.	Mild injection‐site reactions	1	NCT03934307^10^
**ASO/liver**									
Inotersen	ATTR	ATTRv	S.c.	15 months	Improvement in the course of neurologic disease and QOL.	Collection of AEs, clinical laboratory tests, vital signs, ECG, and electroretinography examinations to detect early signs of vitamin A deficiency. Vitamin A supplementation.	Injection site reactions, nausea, headache, glomerulonephritis and thrombocytopenia. Thrombocytopenia and glomerulonephritis were managed with enhanced monitoring.	3	NEURO‐TTR^70^
		ATTR‐CM		2 and 3 years	Safe and effective in inhibiting progression and potentially reversing amyloid burden.	Analysis of platelet count, monitoring of adverse renal effects.	Well‐tolerated safety profile. No signs of glomerulonephritis.	Single‐centre, open‐label study^71^	
Eplontersen	ATTR	ATTRv polyneuropathy and CM	S.c.	65 weeks	Reduced TTR, lessened neuropathy and improved QOL.	TEAEs, discontinuations due to TEAE, thrombocytopenia and glomerulonephritis, injection site reactions, flu‐like symptoms, and TEAEs related to abnormal liver function were monitored. Vitamin A supplementation.	Acceptable safety profile	3	NEURO‐TTRansform^72^
					Stable or improved measures of cardiac structure and function vs. historical placebo.				NEURO‐TTRansform^73^
		ATTR‐CM	S.c.		Ongoing		Ongoing	3	CARDIO‐TTRansform (NCT04136171)
**Anti‐miR/Heart**									
CDR132L	miR‐132	Stable chronic HF	I.v.	6 months	CDR132L was safe and well tolerated and showed indications for cardiac functional improvements.	Platelet counts, laboratory assessments of liver and kidney function, hs‐troponin T, ECG.	Thrombocytopenia was not observed, no signs of hepatic or renal toxicity, no arrythmia	1b	NCT04045405 ^79^
		Acute myocardial infarction		6‐month treatment + 6‐month observation	Ongoing	Clinical laboratory assessment, vital signs, physical examination, and ECGs.	Ongoing	2	HF‐REVERT, NCT05350969^80^
**modRNA/heart**									
AZD8601	VEGFA modRNA	Patients with systolic dysfunction undergoing coronary artery bypass surgery	Epi.c.	6 months	Safety and tolerability endpoints were met (primary endpoint).	Echocardiography to assess haemopericardium and tamponade, AEs from surgery until end of follow‐up and SAEs from informed consent until end of follow‐up.	No deaths or treatment‐related SAEs, no AEs of pyrexia or infection, immune reactions or arrythmias, no negative impact on cardiac function	2	EPICCURE,^83^ NCT03370887
**AAV/heart**									
AAV1/SERCA2a (AAV1)	SERCA2a	Advanced HF	I.c.	12 months	Evidence of biological activity in patients without preexisting anti‐AAV1 antibodies.	Antibodies to AAV1 capsid proteins, clinical laboratory values, vital signs, liver and kidney function.	Acceptable safety profile	½	CUPID^13^
				12 months	Reduction of clinical events and hospitalization times, reduction in NT‐proBNP and improvement in cardiac structure.	Antibodies to AAV1 capsid proteins (exclusion criteria), AEs and SAEs.	Well tolerated, with no untoward effects that can be attributed to AAV1/SERCA2a	2	CUPID^14^
				at least 12 months	Neutral results	AEs, AAV1 neutralizing antibody testing	No safety concerns	2b	CUPID2^85^
				12 months	Trial was terminated prematurely.	AEs, liver and renal markers, monitoring of implantable cardioverter‐defibrillator.	Well tolerated, with no untoward effects that can be attributed to AAV1/SERCA2a	2	AGENT‐HF^15^
				6 months	Trial was terminated prematurely	AEs, laboratory blood test, AAV1 neutralizing antibody testing.	No safety concerns	2a	SERCA‐LVAD^88^
BNP116 (AAV218)	Inhibitor‐1c	Advanced HF	I.c.	12 months + 24 months via telephone questionnaires	Ongoing	AEs, all‐cause mortality, HF hospitalization.	Ongoing	1	NCT04179643
TN‐201 (AAV9)	MYBPC3	HCM	I.v.	5 years	Ongoing	AEs and SAEs	Ongoing	1	MyPEAK‐1, NCT05836259
RP‐A601 (AAVrh.74)	PKP2A	ARVC	I.v.	12 months	Ongoing	TEAEs, SAEs and identification of dose limiting toxicities.	Ongoing	1	PKP2‐ACM, NCT05885412
RP‐A501 (AAV9)	LAMP2B	Danon Disease	I.v.	12 months and 60 months	Ongoing	AEs and SAEs	Ongoing	2	NCT06092034
**CRISPR/Cas9/liver**									
NTLA‐2001	ATTR	ATTRv with polyneuropathy	I.v.	28 days	Mild adverse events and decrease in serum TTR	AEs and laboratory findings Pre‐treatment with glucocorticoid and histamine receptor 1 and 2 blockade to mitigate potential proinflammatory effects after LNP infusion	Mild AEs, no SAEs, coagulation and liver function measures remained within normal limits	1	Interim results of NCT04601051^12^
VERVE‐101	PCSK9	Familial hypercholesterolaemia	I.v.	1 year	Ongoing	TEAEs, SAEs and AEs of special interest	Ongoing	1b	NCT05398029

AE, adverse event; ANGPTL3, angiopoietin‐like 3; ARVC, arrhythmogenic right ventricular cardiomyopathy; ASO, antisense oligonucleotide; ATTR, transthyretin amyloidosis; ATTRv, hereditary transthyretin amyloidosis; CM, cardiomyopathy; CV, cardiovascular; ECG, electrocardiogram; Epi.c., epicardial; GTMP, gene therapy medicinal product; HCM, hypertrophic cardiomyopathy; HDL, high‐density lipoprotein; HF, heart failure; hs, high‐sensitivity; i.c., intracoronary; i.v., intravenous; LAMP2B, lysosome‐associated membrane protein 2b; LDL, low‐density lipoprotein; LNP, lipid nanoparticle; Lp(a), lipoprotein(a); LV, left ventricular; modRNA, modified mRNA; MYBPC3, cardiac myosin‐binding protein C; NT‐proBNP, N‐terminal pro‐B‐type natriuretic peptide; PCSK9, proprotein convertase subtilisin/kexin type 9; PKP2a, plakophilin 2a; QOL, quality of life; SAE, serious adverse event; SERCA2a, sarcoplasmic/endoplasmic reticulum Ca^2+^ ATPase 2a; s.c., subcutaneously; siRNA; small interference RNA; TEAE, treatment‐emergent adverse event; TTR, transthyretin; VEGFA, vascular endothelial growth factor‐A.

### Small interfering RNA

#### Transthyretin amyloid cardiomyopathy

Patisiran is the first siRNA‐based drug approved by the U.S. Food and Drug Administration (FDA) and the European Medicines Agency (EMA) in 2018 for the treatment of polyneuropathy of ATTRv.^55^ ATTR cardiomyopathy is caused by the deposition of the misfolded TTR protein in the myocardium.^56^ Since TTR is produced primarily by the liver, patisiran was encapsulated in lipid nanoparticles covered with apolipoprotein E, which mediated its internalization by hepatocytes via the low‐density lipoprotein (LDL) receptor.^57^ In the phase 3 APOLLO randomized controlled trial, patisiran improved neuropathy scores and gait speed in patients with ATTRv and polyneuropathy.^55^ Non‐invasive pressure–volume analysis of the APOLLO study demonstrated that patisiran may delay progression of left ventricular chamber dysfunction starting at 9 months of therapy.^58^ In a predefined subpopulation of APOLLO, patisiran decreased mean left ventricular wall thickness, global longitudinal strain, *N*‐terminal prohormone of brain natriuretic peptide (NT‐proBNP), and adverse cardiac outcomes compared with placebo at month 18.^59^ In the APOLLO‐B trial, patisiran preserved functional capacity, as assessed by changes in 6‐min walking test, and improved the quality of life after 1 year in patients with wild‐type ATTR (ATTRwt) and ATTRv cardiomyopathy.^60^ However, the FDA rejected the supplemental new drug application for patisiran to treat ATTRwt and ATTRv cardiomyopathy, citing insufficient evidence of clinical benefit.

Vutrisiran is another siRNA that prevents hepatic production of TTR. In contrast to patisiran, vutrisiran is directed to the liver by conjugation to N‐acetylgalactosamine. Vutrisiran significantly improved multiple disease‐relevant outcomes for ATTRv with polyneuropathy in the phase 3 HELIOS‐A trial^61^ and demonstrated evidence of potential benefit on exploratory cardiac parameters in HELIOS‐A patients.^62^ The HELIOS‐B^63^ trial showed that among patients with ATTR‐cardiomyopathy, treatment with vutrisiran led to a lower risk of all‐cause mortality and cardiovascular events than placebo and preserved functional capacity and quality of life.

Overall, siRNA‐based agents targeting TTR production effectively halt neuropathy progression and maintain functional capacity in patients with ATTR‐related neurological and cardiac involvement. Further investigations are warranted to determine whether these agents can improve cardiovascular outcomes in ATTR cardiomyopathy patients and reverse the cardiac structural abnormalities associated with the disease.

#### Risk factors of cardiovascular disease and heart failure

Inclisiran is a siRNA that inhibits the translation of the proprotein convertase subtilisin/kexin type 9 (PCSK9), a serine protease that degrades the LDL receptor, which mediates LDL removal by the liver. Its efficacy in reducing LDL‐cholesterol levels has been demonstrated in the phase 3 ORION‐9 trial in patients with heterozygous familial hypercholesterolaemia^8^, ^9^ and in the subsequent ORION‐10 and ORION‐11 randomized controlled trials.^64^ A patient‐level analysis of the three ORION randomized controlled trials showed a lower incidence of major adverse cardiovascular events in patients treated with inclisiran compared to placebo.^65^

Furthermore, LDL‐cholesterol levels could be reduced by blocking angiopoietin‐like 3, a circulating inhibitor of lipoprotein lipase, the enzyme that clears triglyceride‐rich lipoprotein from the circulation, and endothelial lipase.^66^ Results from the phase 1 AROANG1001 study indicated that angiopoietin‐like 3 silencing with the N‐acetylgalactosamine‐conjugated siRNA ARO‐ANG3 was generally well tolerated and can effectively lower circulating concentrations of atherogenic lipoproteins.^67^ Phase 2/3 trials are required to confirm the LDL reduction along cardiovascular outcome events.

Lipoprotein(a) is another lipoprotein whose levels strongly correlate with the risk of atherosclerotic CVD and cardiovascular events.^68^ The phase 1 OCEAN‐DOSE trial demonstrated that inhibition of hepatic lipoprotein(a) synthesis with the siRNA olpasiran abated lipoprotein(a) levels in patients with established atherosclerotic CVD.^11^ Longer and larger trials are ongoing to determine the effect of olpasiran therapy on CVD outcomes [OCEAN(a)‐Outcomes Trial, NCT05581303].

Finally, knockdown of hepatic angiotensinogen, the precursor of all angiotensins, via the siRNA zilebesiran markedly decreases serum angiotensinogen, the precursor of angiotensin II, and blood pressure for a duration of at least 24 weeks.^10^ Zilebesiran is being further evaluated as a potential treatment for hypertension in two phase 2 studies: KARDIA‐1 and KARDIA‐2.^69^

### Antisense oligonucleotides

Inotersen is a 2′‐O‐methoxyethyl‐modified ASO that targets the 3′ untranslated region of human TTR mRNA, both wt and variant. In the NEURO‐TTR phase 3 trial, inotersen improved the course of neurologic disease and quality of life of patients with ATTRv.^70^ However, several adverse events occurred in the treatment group including injection site reactions, nausea, headache, glomerulonephritis and thrombocytopenia. For this reason, frequent monitoring of renal function and platelet count is advised during treatment.^70^ A small single‐centre, open‐label study involving patients with ATTRwt‐ or ATTRv cardiomyopathy and New York Heart Association (NYHA) class I–III showed a reduction in left ventricular mass and an improvement in exercise tolerance at a 2‐ and 3‐year follow‐up.^71^ A further study is ongoing (NCT03702829).

Eplontersen is a novel ASO formulation that reduced serum TTR, lessened neuropathy impairment, and improved quality of life compared to a historical placebo in ATTRv polyneuropathy patients in the phase 3 NEURO‐TTRansform trial.^72^ Eplontersen was associated with stable or improved measures of cardiac structure and function in patients with ATTRv polyneuropathy and cardiomyopathy compared to historical placebo.^73^ The CARDIO‐TTRansform trial continues to investigate the effect of eplontersen on ATTR cardiomyopathy (NCT04136171). The primary endpoint is the change from baseline in the 6‐min walk test distance at week 65, measuring physical function.

### Anti‐miRNA

The therapeutic potential of targeting miRNAs in CVD is being extensively investigated in clinical trials. These trials aim to harness the potential of miRNAs as biomarkers for early diagnosis, prognosis, and as therapeutic targets. Various approaches are being explored,^74^, ^75^ including the use of miRNA mimics to restore miRNA levels, be it at lesser extent, and anti‐miRNA oligonucleotides to inhibit the activity of disease‐associated miRNAs. Furthermore, nanoparticle‐mediated delivery systems are being developed to improve the targeted delivery of miRNA‐based therapeutics. Based on *in vitro* and *in vivo* mode‐of‐action studies revealing reversal of cardiomyocyte hypertrophy, normalization of autophagy and Ca^2+^ signalling, and reduction of cardiac fibrosis following treatment with the miR‐132 inhibitor CDR132L,^76^, ^77^, ^78^ CDR132L was tested in a first clinical study patients with stable chronic heart failure. In this study, CDR132L proved to be safe and well tolerated and already showed indications for therapeutic efficacy such as NT‐proBNP reductions in addition to standard of care treatment.^79^ A phase 2 study testing CDR132L in 280 patients with acute myocardial infarction and reduced ejection fraction (HF‐REVERT) is ongoing (NCT05350969).^80^ Primary outcome measure is the change in ejection fraction.

### Modified mRNAs

ModRNA for vascular endothelial growth factor‐A has been tested for the treatment of heart failure.^81^ A safety and tolerability randomized phase 1 clinical trial was performed in patients with type 2 diabetes mellitus and no other disease.^82^ Intradermal injection of vascular endothelial growth factor‐A modmRNA transiently increased the local expression of vascular endothelial growth factor‐A protein and enhanced skin blood flow without relevant adverse side effects. A small, randomized, double‐blind phase 2a study (EPICCURE) evaluating the safety and exploratory efficacy of a naked vascular endothelial growth factor‐A modRNA (AZD8601; 3 mg/patient) in patients with systolic dysfunction undergoing coronary artery bypass surgery has been completed.^83^ Seven patients received 30 epicardial injections of AZD8601 targeting ischaemic but viable myocardium identified by positron emission tomography, while four patients were injected with a placebo. The therapy proved to be safe and showed preliminary subjective effects in patients, but the sponsor decided to discontinue its development in 2022.

### Adeno‐associated viral vectors

The first phase 1/2 clinical trial in 2009 for the treatment of heart failure using AAV was based on an AAV1 vector that expressed the sarcoplasmic/endoplasmic reticulum Ca^2+^ ATPase 2a (SERCA2a) cDNA, which was administered safely through intracoronary infusion.^13^ The rationale for this trial was the observation that SERCA2a levels were lower in failing hearts and that overexpression of SERCA2a improved heart function in both small and large animals.^84^ This first trial was followed in 2011 by a phase 2, double‐blinded, randomized, placebo‐controlled, multicentre study (the CUPID trial) with 39 patients with severe heart failure.^14^ The results of this study demonstrated that the treatment was safe and led to significant improvement in cardiac function and a reduction in hospitalizations. Based on these findings, a larger, phase 2b, randomized, double‐blind, multicentre trial, the CUPID2 study, was conducted in 250 patients with stable symptomatic heart failure of both ischaemic and non‐ischaemic aetiology. Primary endpoint was the composite of recurrent heart failure‐related events, and safety parameters included immune response monitoring (antibody, innate immunity and T‐cell response to AAV1 vector) and viral shedding with detection of AAV1 vector DNA in bodily fluids. This trial, however, generated neutral results.^85^ The possible reasons for the neutral results of CUPID2 have been extensively reviewed.^15^, ^86^ The most likely explanations are the low efficiency of myocardial transduction, possibly related to the relatively low dose of vector administered (1 × 10^13^ AAV1.SERCA2a viral particles) and the suboptimal cardiomyocyte tropism of the AAV1 serotype, and the unaltered SERCA2a levels in human heart failure.^87^ Two other related studies using the same vector – the AGENT‐HF study, which evaluated efficacy in patients with left ventricular assist devices^15^ and the SERCA‐LVAD study,^88^ which studied left ventricular remodelling – were prematurely terminated in 2016 as the sponsor suspended further enrolment following neutral results of the CUPID2 outcome trial.

A cardiotropic AAV variant (AAV2i8, also known as BNP116^89^) is currently being tested in a phase 1 clinical study in which a vector carrying the cDNA for a constitutively active form of inhibitor‐1c is administered intracoronary (NCT04179643), and studied for its safety and tolerability in heart failure patients. Work in small and large animals has shown that inhibitor‐1c inhibits the protein phosphatase‐1, which dephosphorylates phospholamban (PLN) and thus blocks SERCA2a. Thus, the ultimate effect of this gene therapy approach is to increase SERCA2a levels and restore β‐adrenergic stimulation.^90^ In addition, clinical trials with AAV carrying cardiac myosin‐binding protein C (*MYBPC3*) in hypertrophic cardiomyopathy (HCM), lysosome‐associated membrane protein 2b (*LAMP2b*) in Danon disease and plakophilin 2a (*PKP2A*) in arrhythmogenic right ventricular cardiomyopathy (ARVC) patients are currently ongoing (see below, *Table* *1*). These phase 1 trials using AAV vectors have safety and tolerability as primary outcome, including immunogenicity and viral shedding as safety measures. In AAV gene therapy studies for liver diseases, hepatotoxicity is the most common adverse event,^91^ and therefore, despite using AAV vectors with cardiac specific promotors, liver toxicity should and will be closely monitored.

### CRISPR‐Cas9

The first CRISPR/Cas9‐based treatment approved by regulatory authorities in late 2023 was a therapy for sickle cell disease and β‐thalassemia to be administered *ex vivo* to hematopoietic stem cells before bone marrow transplantation.^92^ Despite the enthusiasm in this field and the increasing number of CRISPR‐based treatments in clinical trials each year, adapting CRISPR/Cas9 technologies to *in vivo* applications for organs other than the liver remains a challenge. One of the best known examples of successful CRISPR/Cas9‐based *in vivo* gene editing in humans is the treatment of ATTR.^12^, ^93^ A liver‐targeted lipid nanoparticle delivery system called NTLA‐2001, administered by intravenous infusion, decreased serum TTR protein levels up to 87% in six patients with ATTRv.^12^ In the follow‐up of this trial (NCT04601051), a single intravenous infusion of NTLA‐2001 significantly reduced abnormal TTR levels in ATTR cardiomyopathy patients.^94^ Still, the primary outcome of these phase 1 trial include safety and tolerability, and further studies are needed for proving their efficacy.

Another example of liver‐directed CRISPR‐editing using lipid nanoparticles is the base‐editing of PCSK9. Supported by pre‐clinical evidence in mice and macaques^95^, ^96^ showing stable reduction of plasma PCSK9 and LDL‐cholesterol levels without adverse side effects, this base‐editing technology is currently being evaluated in a phase 1b trial in patients with established atherosclerotic CVD due to heterozygous familial hypercholesterolemia (NCT05398029), with safety and tolerability as primary outcomes, with therapeutic efficacy as secondary endpoint. Distinct safety measures for CRIPR‐editing includes (i) detailed assessment of potential off‐target editing to ensure that base editing is confined to the targeted gene without unintended modifications elsewhere in the genome; (ii) evaluation of immune responses against the components of the gene‐editing system, including any potential immune reaction to the delivery vector.

## What are the future perspectives for the treatment of genetic cardiomyopathies?

**Figure 2:**
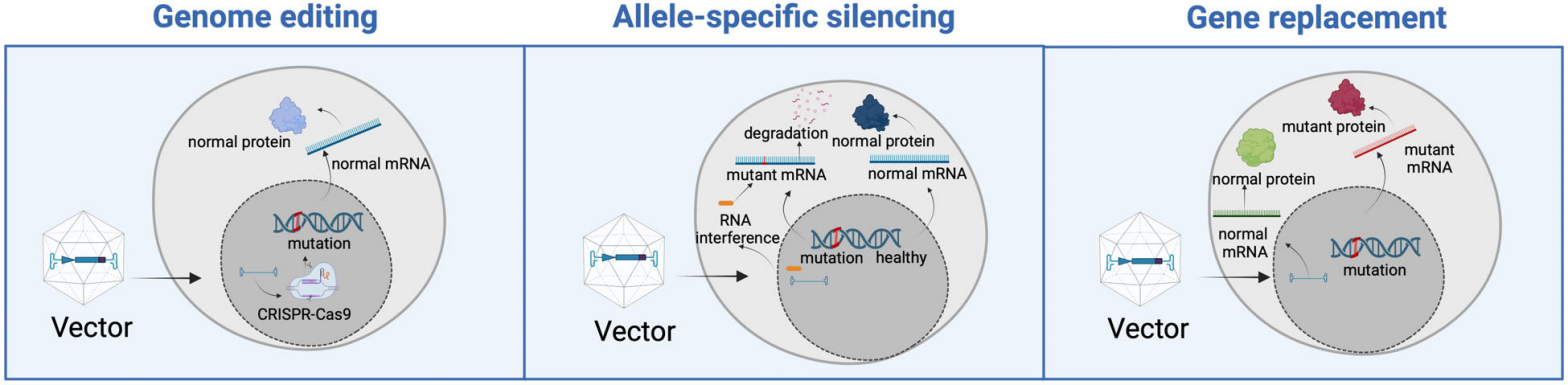
Main strategies used in gene therapy for inherited cardiomyopathies: allele‐specific silencing, gene replacement and gene editing. Created in BioRender. Van Linthout S. (2024) BioRender.com/a38k263

Cardiomyopathies are either inherited (genetic/familial) and/or acquired. They can also be accelerated by disease modifiers. They are a heterogeneous group of diseases and major causes of heart failure. Dilated cardiomyopathy has an estimated prevalence of 1 in 250 to 1 in 2500 of the general population, HCM ranges between 1 in 250 to 1 in 500, and ARVC is estimated to be present in around 1 in 1000 to 1 in 5000 people.^97^, ^98^, ^99^ The current pharmacological treatment of heart failure in cardiomyopathy patients does not significantly differ from general heart failure management.^100^ Phenotype‐ or genotype‐specific drugs are under investigation, with mavacamten being the first disease‐specific drug for inherited HCM, with the exception of ATTRv. In a phase 3, randomized, double‐blind, placebo‐controlled trial (EXPLORER‐HCM), treatment with mavacamten improved exercise capacity, left ventricular outflow tract obstruction, NYHA functional class, and health status in patients with obstructive HCM.^101^ Mavacamten selectively binds to the myosin motor domain, reducing the ATPase activity of myosin. This inhibition decreases the interaction between myosin heads and actin filaments, leading to a reduction in the force of cardiac muscle contraction. As such, mavacamten stabilizes myosin heads in a ‘super‐relaxed’ state, a low‐energy conformation. Gene‐specific treatments, based upon molecular profile or specific cellular, metabolic, contractile, immune or pro‐fibrotic pathways hold therefore great promise, but require further molecular studies to understand the complex mechanisms leading from genetic variants to disease manifestation. Nevertheless, the most logical way to cure an inherited cardiomyopathy would certainly be by gene silencing, replacement or correction (*Figure* *2*), depending on the type of disease‐causing genetic variant (poison peptide or loss‐of‐function). Still, these approaches – in particular AAV and CRISPR – as described in this review still have major challenges in safety, immunogenicity, aspecific gene editing, and long‐term efficacy.

### Silencing gene therapy

Allele‐specific silencing with siRNA or ASO can be used to silence a poison gene product from a mutated allele. In a homozygous *PLN R14del*‐associated cardiomyopathy mouse model, administration of PLN‐ASO led to a dose‐dependent reduction in PLN. Furthermore, this PLN‐ASO prevented PLN protein aggregation, reduced cardiac dysfunction, and increased survival rate in mice.^102^, ^103^ In a *Mybpc3*‐targeted knock‐in mouse model of HCM, skipping of a mutated exon 6 in the *Mybpc3* gene utilizing AAV‐mediated ASO transfer resulted in decreased levels of incorrectly spliced mRNA, restored cardiac function, and halted the development of left ventricular hypertrophy.^104^ Similarly, allele‐specific silencing of mutant *Myh6* transcripts via AAV‐mediated siRNA suppressed HCM in R403Q heterozygous mice.^105^

### Gene replacement therapy

Gene replacement therapy consists of the introduction of a fully functioning gene to replace a loss‐of‐function genetic variant in target cells. Loss‐of‐function variants are non‐sense, splice, or insertion‐deletions causing a frameshift and a premature termination codon (commonly referred to as ‘truncating’ variants, even though the truncated protein is rarely present, due to active degradation of the mutant transcript by the nonsense‐mediated RNA decay).^106^, ^107^ Loss‐of‐function variants are common causes of cardiomyopathy in several genes involved in all cardiomyopathies, HCM (*MYBPC3*)^108^, dilated cardiomyopathy (*titin* [*TTN*]),^109^ Bcl‐2–associated athanogene 3 (*BAG3*),^110^ filamin C (*FLNC*),^111^ desmoplakin (*DSP*)^112^ and ARVC (*PKP2*).^113^

Gene replacement therapy has been tested in mouse or human cellular models of HCM (*MYBPC3*) and ARVC (*PKP2*). It is worth noting that gene replacement therapy may also be effective for missense variants in sarcomeric proteins, because of stoichiometric replacement of endogenous protein. For *MYBPC3*, it has been shown that AAV9‐*Mybpc3* prevented the development of the cardiomyopathic phenotype in *Mybpc3*‐deficient mice^114^ and rescued cell hypertrophy in patient‐derived induced pluripotent stem cell (iPSC)‐derived cardiomyocytes.^115^ These proof‐of‐concept studies motivated the development of a *Mybpc3* gene therapy medicinal product, TN‐201, which is now tested in a phase 1 clinical study (NCT05836259) (*Table* *1*). Similarly, *Pkp2* gene therapy has been shown to reduce ventricular arrhythmias, reverse right ventricular remodelling, improve cardiac function and prolong survival in a *Pkp2*‐deficient mouse model of ARVC.^116^ A gene therapy medicinal product, RP‐A601 (AAVrh.14‐PKP2a), is now evaluated in a phase 1 trial (NCT05885412) (*Table* *1*).

An additional gene replacement therapy relevant to cardiomyopathy is RP‐A501. It is designed to address Danon disease, a rare X‐linked monogenic condition, caused by mutations in the *LAMP2B* gene that often leads to heart failure due to massive left ventricular hypertrophy, and in male patients to frequent death in adolescence or earlier.^117^ Evaluation of RP‐A501, an AAV with a normal copy of the human *LAMP2B* gene (AAV9:LAMP2B), is currently ongoing in a phase 2 trial (NCT06092034) (*Table* *1*).

### Gene editing therapies

In the context of heart failure, studies have now shown *in vitro* (hiPSC‐derived cardiomyocytes) and *in vivo* efficacy of base editing by correcting specific pathogenic variants for lamin A/C (*LMNA*), RNA binding motif protein 20 (*RBM20*), myosin heavy chain 6 (*MYH6*) and myosin heavy chain 7 (*MYH7*) in relevant disease models.^118^, ^119^, ^120^, ^121^, ^122^ Beyond correcting disease‐causing variants, base editing can also be used for exon skipping, which has shown therapeutical relevance particularly in the context of Duchenne muscular dystrophy.^123^, ^124^

Prime editing is of particular interest for modelling and correcting variants associated with cardiac disease because it can target a wide range of single nucleotide variants and more complicated types of genetic alterations (such as short deletions or insertions).^125^ While multiple studies have successfully used prime editing in hiPSC‐derived cardiomyocyte cultures^123^, ^126^, ^127^ and *in vivo*,^119^ this technology so far remains less developed due to lower efficiency rates.

## Safety

Many advances have been made in the management of acute effects related to gene therapies. However, the potential for delayed effects remains a concern, particularly since most gene therapies are designed to achieve permanent or long‐lasting effects in the human body, and this inherently increases the risk of delayed adverse events.^128^ Published evidence of long‐term safety (>1 year) in pre‐clinical setting is rare and has been shown for AAV‐mediated gene therapy outside the cardiovascular field, in the canine model of haemophilia A after a median follow‐up of 10.8 years.^129^ In the cardiovascular field, follow‐up of gene therapy for 6 months or 1 year in large animal models including pigs and non‐human primates have been reported.^12^, ^78^ As far as the safety profile of cardiovascular gene therapy in patients is concerned, trials have shown a very good safety profile even after 10‐year follow‐up for AAV gene transfer overexpressing a gene of interest.^130^, ^131^, ^132^ In general, safety concerns of gene therapy should be differentiated depending on the gene therapy modality used. In 2020 the FDA updated guidelines on the design of long‐term follow‐up studies for the collection of data on delayed adverse events following the administration of a gene therapy product. For studies using integrating vectors and genome‐editing products, patients should be followed up for at least 15 years, while for AAV, a minimum 5‐year follow‐up period is recommended. These recommendations are aligned with those suggested by European Union regulators.^128^

An overview of adverse side effects of gene therapy modalities and mitigation strategies is listed below and in *Table* *2*.

**Table 2:** Adverse effects of gene therapy modalities

Gene therapy modality	Adverse effects	Mitigation strategies
**Targeted gene silencing and regulation therapy**		
Oligonucleotides^133^, ^134^ (ASO, siRNA, miRNA)	Off‐target effects	Selection of sequences with high target specificity and minimal off‐target potential via bio‐informatics. Study of endogenous RNA modifications, RNA structure and RNA‐binding protein binding sites at or near the target sequence to increase the understanding of RNA biology at the target site of interest. Use of chemically modified nucleotides to increase specificity. Pre‐clinical testing.
	Immunogenicity	Use of biocompatible and biodegradable materials for RNA delivery minimizing long‐term effects. Targeted delivery: development of targeting ligands on delivery systems to ensure specific delivery to the affected tissues, reducing systemic exposure. Chemical modifications to the oligonucleotide to reduce immunogenicity. RNA molecule encapsulation in lipid nanoparticles to shield them from immune surveillance. Dose adjustment to minimize immune activation while maintaining therapeutic efficacy. Anti‐inflammatory drug administration before or after the treatment. Pre‐clinical testing.
	Liver toxicity	Monitoring of liver toxicity. Delivery optimization. Pre‐clinical testing.
	Renal toxicity	Monitoring of renal toxicity. Delivery optimization. Proper hydration before and during treatment to reduce renal accumulation. Pre‐clinical testing.
	Injection site reactions (pain, swelling, or redness)	Documentation of local site reactions. Delivery optimization. Pre‐clinical testing.
	Thrombocytopenia and coagulation disorders	Documentation of systemic immune responses and monitoring of leucocyte and platelet counts, coagulation, temperature and flu‐like symptoms. In case of thrombosis, search for secondary causes. Monitoring of bleeding history, coagulation molecules, haemoglobin and platelet count before therapy application. Delivery optimization. Dose schedule optimization by increasing the time intervals between drug administration regimes to enable the platelet count and the coagulation system to recover before the next treatment. Therapy discontinuation if therapy interferes with blood cell production. Pre‐clinical testing.
	Hypersensitivity and allergic reactions	Pre‐treatment screening for allergies or hypersensitivity to any components of the RNA therapeutic or its delivery system. Documentation of systemic immune responses and monitoring of leucocyte counts, temperature and flu‐like symptoms. For patients with mild allergies, gradual dose escalation or pre‐treatment with antihistamines or corticosteroids to mitigate hypersensitivity reactions. Regularly monitoring for signs of autoimmunity during and after treatment to intervene early if symptoms arise. Therapy discontinuation in case of involvement of a systemic adaptive immune response and consideration for alternative delivery systems if hypersensitivity to a specific delivery vehicle is confirmed.
	CNS effects (headaches, mood alterations, or dizziness)	Documentation of CNS effects. Presentation to a neurology specialist in case of symptom persistence. Design RNA therapeutics with restricted ability to cross the blood–brain barrier unless CNS targeting is intended. Discontinuation in case of drug penetrations across the blood–brain barrier. Pre‐clinical testing.
**Targeted gene repair therapy**		
AAV^135^, ^136^, ^137^, ^138^	Immunogenicity	Screening of pre‐existing neutralizing antibodies against AAV. Immunosuppressive (pre‐) treatment. Vector design optimization and shielding to escape the immune system surveillance. Preclinical testing. Regularly monitoring for signs of autoimmunity during and after treatment to intervene early if symptoms arise.
	Liver toxicity	Monitoring of liver function Development of AAV variants for liver de‐targeting and improved cardiac targeting. Use of engineered nanoparticles to promote cardiac tropism. Preclinical testing.
	Insertional mutagenesis	Long‐term follow‐up studies, including genetic monitoring of vector‐treated tissues.
**Precision gene editing therapy**		
CRISPR‐Cas9^139^, ^140^, ^141^, ^142^, ^143^, ^144^, ^145^, ^146^, ^154^	Off‐target effects leading to mutations and DNA damage	Improved Guide RNA Design: design of highly specific guide RNAs with optimized algorithms to reduce off‐target effects. High‐fidelity Cas9 variants: use of engineered Cas9 variants with reduced off‐target activity to minimize off‐target cuts. Chemical modifications of CRISPR/Cas9 *In silico* prediction tools to predict on‐target efficiency and on‐ and off‐target activity Whole exome sequencing as a diagnostic tool to detect off‐target effects, at least in preclinical setting. Use of transient expression of Cas9 delivery systems. Use of self‐limiting vectors that are biodegraded after gene delivery.
	Immunogenicity	Pre‐treatment with immunosuppressive drugs to reduce immune reactions against Cas9, which is a bacterial protein and may trigger an immune response. Chemical modifications to the guide RNA oligonucleotide to reduce immunogenicity. Monitoring of systemic immune responses. Anti‐inflammatory treatment.
	Genomic instability	Comprehensive genomic screening including whole‐genome sequencing. Use of base‐editing or prime‐editing strategies that are less likely to cause large genomic alterations. Use of tissue‐specific promoters to avoid systemic effects. Use of targeted delivery systems.
	Ethical concerns in case of germline editing	Strict regulation and therapy administration monitoring Public engagement and transparency.

AAD, adeno‐associated virus; ASO, antisense oligonucleotide; CNS, central nervous system; miRNA, microRNA; siRNA, small interfering RNA.

### Adverse side effects of oligonucleotides

While oligonucleotide therapies offer exciting possibilities for novel therapeutic approaches, they are not free of potential adverse side effects,^133^, ^134^ including those listed in *Table* *2*. In general, the occurrence and severity of these side effects can vary depending on the specific oligonucleotide, its mode of delivery, and individual patient factors. It is important that potential adverse effects are intensively investigated during the safety and toxicological evaluation in the pre‐clinical phase. Clinical trials are crucial to thoroughly assess and manage the safety profile of oligonucleotide therapies and ensure their optimal and responsible use in the medical field.

### Adverse side effects of adeno‐associated viral vectors

Immunogenicity is a significant concern with AAV‐based gene therapies due to the body's immune response to the viral vector (reviewed in references^135^, ^136^). Many individuals have been exposed to wild‐type AAVs, leading to pre‐existing antibodies that can neutralize the vector, reducing its effectiveness. AAV vectors can trigger innate immune responses shortly after administration, including the activation of complement pathways and the inflammasome.^137^ The capsid proteins of AAV can be recognized by the adaptive immune system, leading to the activation of T cells that may attack transduced cells, reducing gene expression and potentially causing tissue (liver/cardiac) damage. Therefore, patients will get immunosuppressive treatment during 2–3 months, favouring fluid retention, which may be challenging in heart failure patients.

Another important concern is the liver toxicity as AAV vectors accumulate in the liver.^135^ Higher doses of AAV vectors increase the likelihood of hepatotoxicity, which can manifest as elevated liver enzymes, inflammation, and, in severe cases, acute liver failure, resulting in death. The immune responses against the AAV capsid or transgene can lead to cytotoxicity in liver cells, causing inflammation and tissue injury. Mitigation strategies include careful dose selection to balance therapeutic benefits and minimize liver toxicity, provide immunosuppression to minimize the immune response in the liver. Regular monitoring of liver function tests to detect early signs of hepatotoxicity is therefore required. Specific AAV capsid variants have been generated to improve liver de‐targeting and cardiac targeting as well as engineered nanoparticles to promote cardiac tropism of AAV vectors (see up and below).^138^

### Adverse side effects of CRISPR‐Cas9

A major concern with CRISPR‐Cas9 gene editing is the off‐target effects of this gene‐editing tool, where unintended editing events of unrelated genes can lead to mutations and DNA damage.^139^ Off‐target mutations occur more frequently than expected mutations when CRISPR/Cas9 is used.^140^ These off‐target mutations not only disrupt the functionality of unrelated genes, but can also induce genomic instability.^140^ Recent findings reveal that the expression of Cas9 protein in pigs significantly increases genomic damages, induces transcriptome changes, and enriched genomic mutations with prolonged expression of Cas9 resulting in growth retardation and alterations of key tumor‐driving genes in the absence of direct tumour development.^141^ Various modifications of the CRISPR/Cas9 system have been proposed to address this issue. *In silico* prediction tools are used to predict the on‐target efficiency and enable the development of improved algorithms to predict on‐ and off‐target activity.^142^ Additionally, chemical modifications to guide RNA have been proposed to minimize the probability of off‐target DNA cuts.^143^ Regulating the activity of Cas9 is also crucial for safe and efficient editing. Optimizing Cas9 by engineering high‐fidelity variants, adding cytosine extensions to single‐guide RNAs, or using Cas9‐targeting guide RNA to restrict CRISPR expression have been shown to reduce off‐target effects.^144^, ^145^, ^146^ The immunogenicity of the CRISPR‐Cas9 system has also been reported as a detrimental feature. It may lead to frequent activation of p53, DNA damage, large‐scale on‐target genomic deletion and chromosomal rearrangement.^147^, ^148^, ^149^, ^150^

The half‐life of the editors in the cells has a major influence on the activity and reliability of gene editing. The methods by which Cas9/single guide RNA is introduced into target cells, such as plasmid transfection, ribonucleoprotein, electroporation, or viral transduction, may affect its off‐target effects.^151^ AAV and lipid nanoparticles are commonly used vectors for *in vivo* gene therapy. AAV9 is effective in infecting the hearts of mice and large mammals,^54^ but it may accumulate off‐target mutations over time due to its long‐lasting expression.^152^, ^153^ On the other hand, lipid nanoparticles can be rapidly degraded *in vivo*, making them popular for gene editing.^154^ In this regard, lipid nanoparticle‐encapsulating Cas9/single guide RNA targeting TTR has been shown to be a safe and efficient form of delivering gene editing tools in patients.^12^ However, it is important to point out that these reflect interim results until day 28 post application, whereas patients will be long‐term followed up.

## Design of clinical studies and regulatory strategies

Gene therapies are still in their infancy. To date 26 gene therapy products have been approved worldwide.^155^ Guidelines by the regulatory agencies FDA^156^ and EMA are expected to further accelerate clinical application in this area and provide greater clarity for manufacturers and healthcare professionals.^157^ The role of the pharmaceutical industry in securing the investments needed for the expensive regulatory trials allowing full clinical development (from phase 1 to 4) cannot be ignored in this process.^158^ Therefore, it is advised to be aware – even in the early stages of research – not only of the therapeutic potential of a novel therapy, but also of its potential to become both realistic and attractive to the pharmaceutical industry.^159^ The requirements for such trials – and the timeframe for the development phase – can be considerably reduced for rare diseases and orphan drugs, or if the clinical phase can be optimized through innovative features such as adaptive designs, umbrella or basket designs, or n‐of‐1 trials.^158^ In the cardiology field, however, the traditional phase 1, 2, 3 design is still followed in most cases. However, it is acceptable to conduct phase 1 trials directly in patients with heart failure or the specific disease phenotype of interest without a preliminary phase in healthy volunteers, as was done in a recent antisense RNA trial in heart failure patients.^79^ This is one way of making drug development faster and more efficient without compromising on safety. The safety and efficacy requirements defined by the regulatory authorities and guideline committees for the approval and indications of drugs must of course also apply for novel gene therapies.

## Future new modalities for viral and non‐viral therapy

### Cardiac targeting

In contrast to the success in the development of AAV variants with improved cardiac targeting and liver de‐targeting (see before),^86^, ^160^, ^161^ specific targeting of cardiomyocytes and liver detargeting has been more problematic so far for non‐viral RNA delivery. This is due to the fact that there is no molecule like N‐acetylgalactosamine in the liver for any cardiac cell type. Most of the systemically administered nucleic acid‐containing nanoparticles are phagocytosed by mononuclear cells in the spleen, lymph nodes and bone marrow or end up in the liver, and only a small proportion is retained in the heart. Cardiac retention, however, is increased after acute damage and inflammation, such as myocardial infarction. This is thanks to the transient vascular permeability and retention effect, by which relatively large fenestrations form between endothelial cells, permitting nanosized particles to extravasate from the circulation into the inflamed tissue.^162^

A number of cardiac‐specific peptides have been identified that can be conjugated by nanoparticles to enhance cardiac targeting. These have been identified by screening phage display libraries or are ligands for or antibodies against endogenous cardiomyocyte or endothelial receptors.^5^ Recent evidence demonstrates that cargo‐less, safe poly (lactic‐co‐glycolic acid) particles can drastically improve heart delivery of AAVs and nanoparticles based on the interaction of the glucose–GLUT axis, circumventing active targeting (e.g. via epitope recognition) or serotype engineering for AAV.^138^

While these targeting technologies are promising but do not yet appear to be mature for clinical application, with liver detargeting remaining a major unsolved issue, the heart can however be reached through direct intravascular catheterization for local vector or RNA administration. In the case of vascular infusion, this can be done anterograde through the coronary arteries or retrograde from the coronary sinus, or, in the case of intramyocardial injection, trans‐endocardial from the left ventricle.^86^

### Delivery route

Choosing the best approach for cardiac gene therapy delivery – whether intracoronary, intravenous, intramyocardial or epicardial – depends on factors like the target cardiac disease, desired gene expression level, and invasiveness. Intracoronary delivery is often favoured for its ability to target the heart with less invasiveness compared to direct intramyocardial injections, and higher transduction efficacy in the target area and a reduced off‐target contamination compared to systemic intravenous application. Antegrade delivery however, may be impaired by coronary heart disease, such as stenosis or occlusion of a coronary artery. Coronary veins appear not to be affected and might therefore be the preferable application route for gene therapy.^163^ For an effective and safe retrograde application, selective catheterization of the coronary vein draining the target area is necessary. A selective pressure regulation of retroinfusion enhances safety and seems to be a favourable approach for gene therapy transduction in combination with reduced systemic contamination.^163^ Intracoronary application paired with proximal balloon occlusion to limit competitive flow, results in efficient uptake of both AAV and similarly sized nanoparticles and allows for delivery to the entire vascular bed of interest and regions of the heart not easily reached via epicardial injection.^164^ Epicardial approaches offer more precise delivery but are also more invasive. Non‐invasive, intratracheal administration is used for gene therapy directed to treat pulmonary hypertension.^165^ For AAV gene therapy, mostly the intravenous route is now being tested in view of the cardiac promotors that allow high cardiac specificity of gene overexpression.^166^

Ultrasound‐assisted gene delivery to the heart is an exciting and emerging methodology showing tremendous advancements in pre‐clinical models to date, reinforcing the idea that translation of contrast echocardiography‐mediated localized cardiac gene delivery is a feasible near‐term objective.^167^ Further investigations in large animal models are warranted to understand the implications of each technique for AAV‐ and nanoparticle‐based therapeutic outcomes.

### RNA editing with CRISPR/Cas9

In addition to targeted DNA mutagenesis, the CRISPR/Cas system can edit various types of RNA, such as miRNA, long‐noncoding RNA, and mRNA.^168^ Aberrant m6A RNA methylation and dysregulated adenosine‐to‐inosine editing, mediated by the enzymes adenosine deaminases acting on RNA (ADARs)^169^, ^170^ contribute to the development of heart failure.^171^, ^172^ The combination of CRISPR/Cas technology with single‐chain m6A methyltransferase or demethyltransferase enables site‐specific insertion or deletion of m6A modifications.^173^ In addition, several innovative RNA editing platforms have been developed to perform targeted RNA base conversions mediated by ADARs.^168^, ^174^, ^175^, ^176^ These RNA editing systems exhibit versatility, high specificity, and efficiency and facilitate the editing of full‐length mRNA transcripts containing disease‐associated point mutations.^177^

RNA editing is considered less risky than DNA editing because it is only transient and may not cause off‐target mutations.^169^ RNA editing/modifying platforms are therefore promising additions to existing CRISPR/Cas systems.

## Conclusion

Advances in gene therapy including the advent of CRISPR genome editing and improved targeting and silencing strategies, have launched a new era of cardiovascular research, enabling targeted therapeutics and improved implementation of precision and personalized medicine. To date, clinical success has been achieved primarily with gene therapies targeting the liver, but there have also been initial breakthroughs in the development of cardiac‐directed therapies for inherited cardiomyopathies in pre‐clinical models. In addition, pivotal clinical trials are currently underway to test cardiac gene therapy for cardiomyopathies. Despite the many advances in the field of gene therapy, there is still much to learn. A better understanding of the consequences of introducing lifelong genetic modifications and the safety profile of these editing techniques will be crucial for the development of gene editing‐based therapies for cardiac conditions. Further improvements in tissue‐specific delivery through chemical modifications, bioconjugation and the use of nanocarriers may ultimately lead to reduced toxicity, lower immunogenicity and better efficacy. With regard to anti‐miRNAs in CVD, further research is needed to uncover the complex regulatory networks involved to ensure the safety and efficacy of miRNA‐based therapies. Overall, gene therapy has the potential to transform treatment strategies aimed at alleviating symptoms and complications into strategies that address the underlying causes of diseases. Further success of this promising but delicate field of future medical treatment will depend on close cooperation between regulatory authorities, the pharmaceutical industry, patients and research institutions.

## Conflict of Interest

M.G. is scientific founder, consultant, member of the Board and equity holder in Purespring Therapeutics, Forcefield Therapeutics and Heqet Therapeutics. L.C. is a member of DiNAQOR Scientific Advisory Board and has shares in DiNAQOR. P.G.P. reports speaker fees from BMS, Pfizer, Bridgebio, Ionis Pharmaceuticals, AstraZeneca, NovoNordisk, Intellia and Alnylam Pharmaceuticals, consulting fees from BMS, Cytokinetics, Rocket Pharmaceuticals, Lexeo, Pfizer, Bridgebio, Daiichi Sankyo, Neuroimmune, Alnylam Pharmaceuticals, AstraZeneca, NovoNordisk, ATTRalus, Intellia, Idoven, General Electric and Alexion, and research/educational support to its institution from Pfizer, Bridgebio, NovoNordisk, AstraZeneca, Intellia and Alnylam Pharmaceuticals. L.R.L. reports speaker fees from BMS, Sanofi, and Alnylam, consulting fees from Novo Nordisk and BMS, and received a research grant from BMS. P.M. is co‐founder, shareholder and CEO of AaviGen GmbH. K.S.B. received research support from Novartis and BionTECH and speaker's honoraria from Novartis. H.S. received consultancy fees from Novartis, Pharmacosmos, AstraZeneca, Daiichi Sankyo, Bayer, Boehringer Ingelheim. T.T. filed and licensed patents in the field of non‐coding RNA based therapeutics; is founder and shareholder of Cardior Pharmaceuticals GmbH; received personal fees for lectures and/or scientific advice from Bayer, Boehringer Ingelheim, Novo Nordisk, Sanofi‐Aventis, Takeda, Amicus Therapeutics. C.G.T. reports honoraria or consultation fees from VivaLyfe, Univers Formazione, Solaris, Summeet, AstraZeneca, Myocardial Solutions, Medtronic; funding from Amgen and MSD; listed as an inventor of two patents related to heart failure, outside the submitted work. C.T. has received speaker fees and/or contributions to congresses from Abbott, Abiomed, AstraZeneca, Bayer, Berlin Chemie, Novartis, Pfizer, and Servier; all outside the submitted work. The UMCG, which employs P.v.d.M., received consultancy fees and/or grants from Novartis, Pharmacosmos, Vifor Pharma, Astra Zeneca, Pfizer, Pharma Nord, BridgeBio, Novo Nordisk, Daiichi Sankyo, Boehringer Ingelheim and Ionis. E.V.R. is a founder of Phlox Therapeutics and Revier Therapeutics and a scientific advisor for Heartbeat.Bio and Tenaya Therapeutics. M.M. received consultancy honoraria from Abbott Structural heart, AstraZeneca, Bayer, Boehringer Ingelheim, Edwards lifeSciences, Novo Nordisk, Roche diagnostics. S.H. receives personal fees for independent scientific advice on early development in the field of heart failure for AstraZeneca, Ribocure, and CSL Behring, and receives research support from AstraZeneca and CSL Behring. All other authors have nothing to disclose.
